# Are Organic Certified Carrots Richer in Health-Promoting Phenolics and Carotenoids than the Conventionally Grown Ones?

**DOI:** 10.3390/molecules27134184

**Published:** 2022-06-29

**Authors:** Dominika Średnicka-Tober, Klaudia Kopczyńska, Rita Góralska-Walczak, Ewelina Hallmann, Marcin Barański, Krystian Marszałek, Renata Kazimierczak

**Affiliations:** 1 Department of Functional and Organic Food, Institute of Human Nutrition Sciences, Warsaw University of Life Sciences, Nowoursynowska 159c, 02-776 Warsaw, Poland; klaudia_kopczynska@sggw.edu.pl (K.K.); rita_goralska_walczak@sggw.edu.pl (R.G.-W.); ewelina_hallmann@sggw.edu.pl (E.H.); renata_kazimierczak@sggw.edu.pl (R.K.); 2 Laboratory of Neurobiology, Nencki Institute of Experimental Biology, Polish Academy of Sciences, Pasteura 3, 02-093 Warsaw, Poland; m.baranski@nencki.edu.pl; 3 Department of Fruit and Vegetable Product Technology, Prof. Wacław Dąbrowski Institute of Agricultural and Food Biotechnology, Rakowiecka 36, 02-532 Warsaw, Poland; krystian.marszalek@ibprs.pl

**Keywords:** carrot, organic production, organic food, bioactive compounds, carotenoids, phenolics, antioxidants

## Abstract

The aim of the present study was to determine the concentrations of polyphenols and carotenoids by means of HPLC/UV-Vis in certified organic and non-organic carrots (*Daucus carota* L.) of two cultivars (Flacoro and Nantejska). The analyzed carrot root samples contained, on average, 4.29 ± 0.83 mg/100g f.w. of carotenoids (mainly *β*-carotene) and 9.09 ± 2.97 mg/100g f.w. of polyphenols, including 4.44 ± 1.42 mg/100g f.w. of phenolic acids and 4.65 ± 1.96 mg/100g f.w. of flavonoids. Significant effects of the production system on the carotenoids (total) and *β*-carotene concentration were found, with higher concentrations of these compounds generally identified in conventionally cultivated roots (4.67 ± 0.88 mg/100g f.w.) vs. organically grown ones (4.08 ± 0.74 mg/100g f.w.). There was a noticeable inter-sample (inter-farm) variation in the concentration of polyphenols in carrot roots. Despite a general trend towards higher concentrations of these compounds in the organic carrots (9.33 ± 3.17 mg/100g f.w.) vs. conventional carrots (8.64 ± 2.58 mg/100g f.w.), and in those of Nantejska (9.60 ± 2.87 mg/100g f.w.) vs. Flacoro (8.46 ± 3.02 mg/100g f.w.) cultivar, no consistent, statistically significant impact of the production system and/or cultivar on the level of these bioactive compounds was identified. More efforts should be encouraged to ensure that organic crops reaching the market consistently contain the expected high levels of health-promoting bioactive compounds, which could be brought through their shelf-life and all processing steps, in order to meet consumers’ expectations and provide the expected health benefits.

## 1. Introduction

Growing concerns about the negative environmental impacts of intensive farming practices used in the conventional food production sector have become, in the last few years, a meaningful driver for the development of alternative, less aggressive systems, including the organic food production system [1]. Organic farming is at the present time broadly considered to be a step towards more environmentally and socially sustainable food production, addressing many of the needs defined by the UN Environment Programme [2,3]. Organic production relies on natural fertilizers, mechanical and biological plant protection, and rich crop rotations [4]. Organic farmers put significant effort into protecting biodiversity on their farms, employing practices capable of maintaining high standards of soil fertility and also limiting the use of natural resources. Despite the fact that some of the sustainability measures of organic food systems remain as points of intensive scientific debate [5], the values of certain organic farming methods have been supported by a significant number of research reports [6].

At the same time, as confirmed in extensive international studies, organically grown crops are less frequently contaminated with pesticide residues [7], and organic food consumption was recently shown to significantly reduce consumers’ exposure to all groups of synthetic chemical pesticides [8]. Moreover, the agronomic practices and inputs used by organic farmers were shown to impact minerals uptake and metabolic processes in plants, resulting in differences in the profiles of plant secondary metabolites [9,10]. In practice, organically grown crops have been suggested to be, on average, up to 60% richer in various health-promoting bioactive phenolics when compared to conventionally cultivated ones [7,11,12,13].

All of these characteristics of organic agriculture and organically produced foods respond to the expectations of an increasing number of consumers searching for safe, high-quality products produced with the use of natural, environmentally friendly means and methods. Therefore, the organic food market, including the sector of fresh fruits and vegetables, is experiencing very dynamic growth globally [1].

These quality and safety aspects of fruit and vegetable production have recently gained attention in light of the current World Health Organization (WHO) recommendations to increase consumption of these food groups to a minimum of 400 g per day [14]. This amount of vegetables and fruits, so long as it is pesticide-free, has been proven to be one of the crucial factors in the prevention of various non-communicable diseases, including cancers, diabetes, obesity, and cardiovascular disease [14,15,16,17]. Thus, taking action to increase the consumption of fruits and vegetables is seen as an important step, not only towards overall food system sustainability but also towards a healthy diet.

It is worth pointing out that, among the published studies on the quality of organic crops, only a very limited number have focused on carrots (*Daucus carota* L.) [7,18]. Carrot is a popular root vegetable, cultivated in most areas of the globe, on close to 1.14 million hectares. Its global annual production reaches nearly 45 million tonnes, with the highest figures recorded in Asia (over 28 million tonnes) and Europe (over 9 million tonnes) [19]. In recent years, the consumption of carrots and carrot-based processed foods, including those organically produced, has been increasing steadily due to the recognition of carrots as a rich source of not only carotenoids and dietary fiber but also other bioactive compounds with proven health-promoting potential [20,21]. Considering the above, carrying out research to investigate the qualities of carrots, especially in alternative, low-input agricultural systems, is of significant potential benefit for producers and consumers. The aim of the present study was, therefore, to analyze and compare the concentrations of the selected groups of bioactive compounds—polyphenols and carotenoids—in the carrots of two common cultivars (Flacoro and Nantejska) grown in both the conventional system and the certified organic system in Poland.

## 2. Results and Discussion

**Figure 1:**
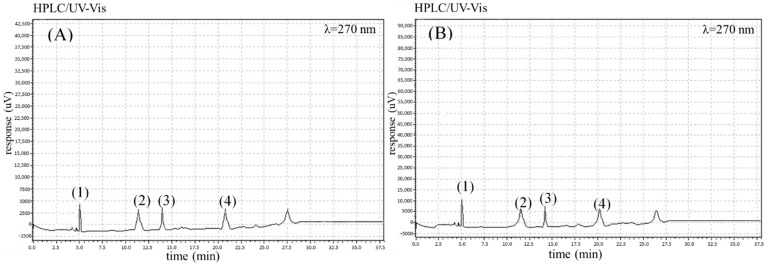
The chromatograms showing the retention time for phenolic acids in the selected organic (**A**) and conventional (**B**) carrot samples: (1) gallic acid, (2) chlorogenic acid, (3) caffeic acid, and (4) *p*-coumaric acid.

**Figure 2:**
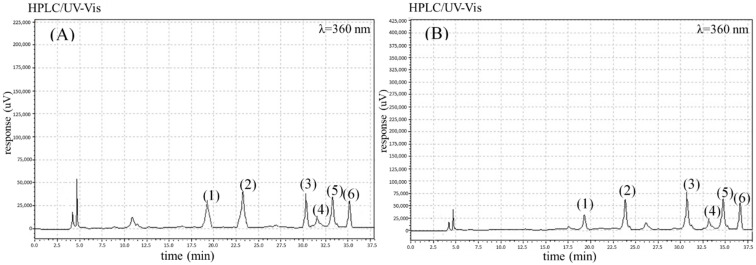
The chromatograms showing the retention time for flavonoids in the selected organic (**A**) and conventional (**B**) carrot samples: (1) quercetin-3-*O*-rutinoside, (2) kaempferol-3-*O*-glucoside, (3) luteolin, (4) apigenin, (5) quercetin-3-*O*-glucoside, and (6) kaempferol.

**Figure 3:**
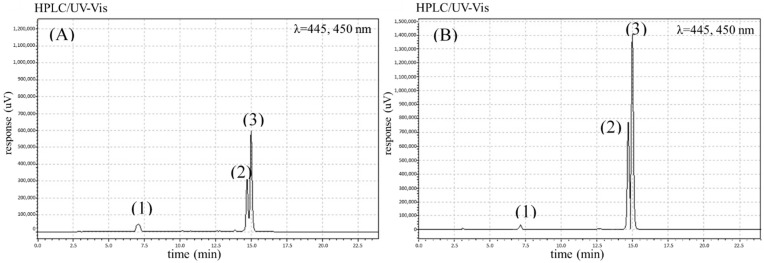
The chromatograms showing the retention time for carotenoids in the selected organic (**A**) and conventional (**B**) carrot samples: (1) lutein, (2) α-carotene, and (3) β-carotene.

The analyses performed permitted the identification of four phenolic acids (chlorogenic, gallic, caffeic and *p*-coumaric acid), six flavonoids (quercetin-3-*O*-rutinoside, quercetin-3-*O*-glycoside, kaempferol, kaempferol-3-*O*-glycoside, luteolin, and apigenin) and three carotenoids (*β*-carotene, *α*-carotene, and lutein) in the carrot root samples studied. The chromatograms for the identified phenolics and carotenoids are shown in Figure 1 (phenolic acids), Figure 2 (flavonoids) and Figure 3 (carotenoids).

The analyzed carrot (*Daucus carota* L., cv. Flacoro and Nantejska) root samples contained, on average, 13.45 ± 1.65% of dry matter (13.73 ± 1.67% in organic vs. 12.95 ± 1.53% in conventional samples), 4.29 ± 0.83 mg/100g f.w. of carotenoids (mainly *β*-carotene) (4.08 ± 0.74 mg/100g f.w. in organic vs. 4.67 ± 0.88 mg/100g f.w. in conventional samples) and 9.09 ± 2.97 mg/100g f.w. of polyphenols (9.33 ± 3.17 mg/100g f.w. in organic vs. 8.64 ± 2.58 mg/100g f.w. in conventional samples), including 4.44 ± 1.42 mg/100g f.w. of phenolic acids (4.52 ± 1.37 mg/100g f.w. in organic vs. 4.30 ± 1.54 mg/100g f.w. in conventional samples) and 4.65 ± 1.96 mg/100g f.w. of flavonoids (4.81 ± 2.15 mg/100g f.w. in organic vs. 4.33 ± 1.55 mg/100g f.w. in conventional samples) (Table 1).

Carotenoids are fat-soluble pigments responsible for the yellow, orange, and red coloring of many fruits and vegetables. They have been shown to have a number of important biological roles, acting not only as provitamins but also as strong health-promoting antioxidants [22]. Kazimierczak et al. have reported higher carotenoids concentrations, including lutein and *β*-carotene, in conventional compared to organic medicinal plants [23]. In our study, a significant effect of the production system on the carotenoids (total) and *β*-carotene concentration in carrots was also found, with higher concentrations of these compounds generally identified in the conventionally cultivated roots. However, in the case of *β*-carotene, a significant interaction between the production system and the cultivar was also noted: The described significant production system effect was evident only for the Nantejska cultivar. At the same time, neither the production system nor the cultivar had an impact on the concentrations of the two other identified carotenoids—lutein and α-carotene—within the carrot roots (Table 1). Chenard et al. [24] and Boskovic-Rakocevic [25] linked the increase in the level of carotenoids (especially *β*-carotene) in parsley and carrot roots to the high rates of easily soluble nitrogen fertilization typical of conventional farming. Similarly, positive correlations between the carotenoids in leaves and nitrogen supply have been previously reported in kale [26], watercress [27] and lettuce [28]. Kaack et al. [29] have confirmed that the synthesis of carotenoids in carrot roots was positively associated with nitrogen availability at the beginning of the growing season. As organically managed soils are often characterized by lower nitrogen availability, one might expect to find reduced levels of carotenoids in organically grown crops.

Another complex and diverse group of phytonutrients identified in carrot roots are phenolics, which are among the most abundant antioxidants in the human diet. The available scientific literature suggests the contribution of these bioactive compounds in the prevention of cancers, neurodegenerative diseases, type 2 diabetes, osteoporosis, and cardiovascular diseases, through and beyond oxidative stress modulation [30,31,32]. The profiles of these biologically active compounds in plants are known to be impacted by genetic, environmental, and agricultural factors, including those related to cultivation and post-harvest practices [33]. According to the largest published meta-analysis comparing the composition of organic vs. non-organic foods, the organically cultivated crops were reported to be, on average, up to 60% richer in phenolics when compared to conventionally cultivated ones [7], even though there was a considerable variation among various crop types and species.

In our study, there was a large inter-sample (inter-farm) variation in the concentrations of polyphenols (including phenolic acids and flavonoids) in the carrot roots; thus, no statistically significant impact of the production system and/or cultivar on the levels of these compounds was identified. However, there was a general trend towards higher concentrations of phenolics in the carrots grown in the organic compared to the conventional system, and in those of the Nantejska compared to the Flacoro cultivar (Table 1). Among the identified phenolic compounds, the only significant effects of the agricultural production system were detected for gallic acid, luteolin, and apigenin contents. The gallic acid concentrations were significantly higher in the organic vs. the conventional carrots (2.11 ± 0.87 mg/100g f.w. vs. 1.27 ± 0.26 mg/100g f.w., respectively), and this was especially evident in the Nantejska cultivar (2.38 ± 1.00 mg/100g f.w. vs. 1.14 ± 0.19 mg/100g f.w.). The apigenin content was also higher in the organic carrots (1.54 ± 1.00 mg/100g f.w.) compared to the conventional carrots (1.05 ± 0.59 mg/100g f.w.). At the same time, the opposite trend was observed in the case of luteolin, which reached, on average, higher concentrations in the conventionally grown carrots (0.47 ± 0.36 mg/100g f.w.) vs. the organically grown carrots (0.31 ± 0.21 mg/100g f.w.) (Table 1). The significant inter-sample heterogeneity observed in the results suggests the importance of additional (environmental and/or agronomic) factors modulating the carrot plants’ metabolism and strongly impacting the carrot root’s composition.

Nitrogen accessibility and irradiation were previously demonstrated to be important agricultural and environmental factors determining polyphenol content in both the organic and the conventionally grown crops [34,35]. However, in our study, the nitrogen availability patterns could not be measured; therefore, we were unable to monitor the relation between this potentially explanatory variable and the levels of phenolic compounds in the tested samples.

Previous research on grapes, wheat, and potatoes has indicated that cultivar selection can be the most significant explanatory factor for polyphenols concentrations [36,37,38]. In a recently published study on wheat, a variety coming from the organic agriculture-focused breeding program contained considerably higher phenolics contents when grown with organic as opposed to synthetic mineral fertilizers, while the type of fertilizer had a marginal impact on the phenolic contents in a variety coming from a conventional agriculture-focused breeding program [35].

Since phenolic compounds are known to participate in the plant’s resistance response to diseases and pests, the exposure of plants to these biotrophic stresses can also result in an increase in phenolics concentrations. Higher disease and pest incidence has been previously described as one of the main reasons for higher concentrations of resistance-related compounds in organic compared to conventional crops [39], even though experimental evidence of such an association is lacking [34,35].

The organically grown and conventionally grown carrot roots tested within the study did not differ significantly in their dry matter content.

**Figure 4:**
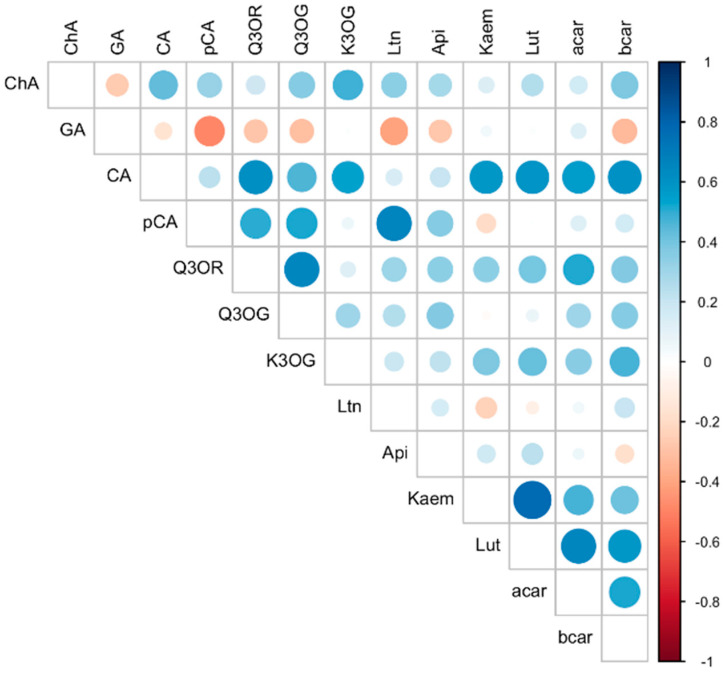
Pearson’s correlations between the concentrations of the individual phenolics and carotenoids identified in the carrot roots. Color (blue/red) and color intensity correspond to the direction and the strength of the correlation, while the circle size corresponds to statistical significance (*p*-value). ChA, chlorogenic acid; GA, gallic acid; CA, caffeic acid; pCA, *p*-coumaric acid; Q3OR, quercetin-3-*O*-rutinoside; Q3OG, quercetin-3-*O*-glycoside; K3OG, kaempferol-3-*O*-glycoside; Ltn, luteolin; Api, apigenin; Kaem, kaempferol; Lut, lutein; αcar, *α*-carotene; βcar, *β*-carotene.

The correlation analysis performed identified a number of significant associations between the concentrations of individual phenolics and carotenoids in the carrot roots (Figure 4).

The strongest positive correlations were identified between concentrations of lutein and kaempferol (r = 0.78, *p* < 0.0001), luteolin and *p*-coumaric acid (r = 0.66, *p* < 0.0001), quercetin-3-*O*-glycoside and quercetin-3-*O*-rutinoside (r = 0.66, *p* < 0.0001), as well as caffeic acid and quercetin-3-*O*-rutinoside (r = 0.62, *p* < 0.0001), *β*-carotene (r = 0.60, *p* < 0.0001), kaempferol (r = 0.59, *p* < 0.0001), lutein (r = 0.59, *p* < 0.0001), *α*-carotene (r = 0.57, *p* < 0.0001), and kaempferol-3-*O*-glycoside (r = 0.55, *p* < 0.0001). Positive correlations were also identified between all of the individual carotenoids: *β*-carotene, *α*-carotene, and lutein (r 0.52–0.66, *p* < 0.0001). Similar positive associations between *β*-carotene and lutein concentrations were previously reported in Canola (*Brassica napus*) oil [40] and lettuce [41]. Moreover, positive correlations between flavonoid and carotenoid contents were previously found in various cultivars of rice (*Oryza sativa* L.) [42]. Negative correlations were identified between the concentrations of gallic acid and luteolin (r = −0.5, *p* < 0.0001) and gallic acid and *p*-coumaric acid (r = −0.4, *p* = 0.001). These overall correlation analysis results could indicate that the synthesis and/or metabolism of these compounds is closely linked and/or regulated by the same agronomic and environmental drivers. A transcriptional regulation network for the biosynthesis of flavonoids and carotenoids was previously demonstrated in ‘Cara Cara’ navel orange [43]. Even though the major genes for carotenoids accumulation differed from the major ones involved in the flavonoid’s biosynthesis, transcriptomic analysis revealed that 24 transcription factors were co-regulators in both pathways, which might play a significant role in the accumulation of carotenoids and flavonoids in the fruit [43].

## 3. Materials and Methods

### 3.1. Study Design and Plant Material

The study was carried out at the Warsaw University of Life Sciences (Poland) in 2015–2016. Carrot roots (*Daucus carota* L., cv. Flacoro and Nantejska) were gained from 7 conventional and 13 organic farms localized in the Mazovia region (Central Poland), all characterized by similar agricultural environment (i.e., similar soil and climate conditions). The random carrot root samples (≥1 kg each) were collected from each farm, transported to the laboratory of the Department of Functional and Organic Food, Warsaw University of Life Sciences, cut into cubes, and freeze-dried (Labconco 2.5 freeze-drier, Labconco Corporation, Kansas City, MI, USA) at a temperature of −40 °C and under a pressure of 10 Pa. Freeze-dried carrot samples were ground in a laboratory mill (A-11 laboratory mill, IKA^®^-Werke GmbH & Co. KG, Staufen im Breisgau, Germany) and stored at −80 °C before further analyses.

### 3.2. Laboratory Analyses

#### 3.2.1. Dry Matter Content

Dry matter content was determined in the samples following the method described by Isaac and Maalekuu (2013) [44]. The samples were weighted and dried at a temperature of 105 °C and at air pressure of 1013 hPa (FP-25W Farma Play dryer, from Farma Play, Marki, Poland) for 48 h, cooled in desiccator at room temperature, and weighed again. The content of dry matter was calculated in g/100 g fresh weight.

#### 3.2.2. Phenolic Compounds Extraction and Determination

The levels of polyphenolic compounds (phenolic acids and flavonoids) were determined by the HPLC/UV-Vis method, as previously described by Hallmann (2012) [45] (equipment: Shimadzu, USA Manufacturing Inc., Canby, OR, USA: two pump LC-20AD, controller CBM-20A, column oven SIL-20AC, spectrometer UV/Vis SPD-20 AV).

The 100 mg samples of freeze-dried carrot powder were mixed with 5 mL of 80% methanol (*v*/*v* aqueous solution), shaken (Micro-Shaker 326 M, Premeo, Marki, Poland), and ultrasonicated (10 min, 30 °C, 5500 Hz). The samples were then centrifuged for 10 min at 5635× *g* and 0 °C. Then, 1 mL of supernatant was placed into vials and used for the analysis. The injection volume was 100 µL. The separation of phenolics was performed on the Synergi Fusion-RP 80i Phenomenex column (250 × 4.60 mm) under gradient conditions with a flow rate of 1 mL/min. The gradients of phase A and phase B were as follows: 10% (*v*/*v*) acetonitrile and ultra-pure water (phase A) and 55% (*v*/*v*) acetonitrile and ultrapure water (phase B). Acidification was performed with 85% ortho-phosphoric acid (a few drops to bring pH to 3.0). The wavelength of 270 nm (for phenolic acids detection) and 360 nm (for flavonoids detection) was used. The external standards of polyphenols (Fluca and Sigma Aldrich) with purities of 95.00–99.99% were used for the identification of compounds. Calibration curves were prepared individually for each of the compounds. For this purpose, pure standards were dissolved in 80% methanol. Standard concentrations were as follows: gallic acid 10 µg/mL, chlorogenic acid 12 µg/mL, caffeic acid 2 µg/mL, *p*-coumaric acid 2 µg/mL, quercetin-3-*O*-rutinoside 15 µg/mL, kaempferol-3-*O*-glycoside 15 µg/mL, luteolin 10 µg/mL, quercetin-3-*O*-glycoside 12 µg/mL, apigenin 19 µg/mL, and kaempferol 15 µg/mL. Each standard was injected five times—every time with doubled volume compared to the previous one. Standard curves were developed based on the outcomes of the described procedure.

#### 3.2.3. Carotenoids Extraction and Determination

The method published by Collera-Zuniga et al. (2005) [46] and also described in the paper by Hallmann and Rembiałkowska (2012) [47] was implemented (with small modifications) for the determination of the carotenoids in the carrot roots. The analysis was performed with the Shimadzu equipment characterized in the previous section.

The freeze-dried carrot samples (100 mg) were mixed with acetone (5 mL). Then, samples were ultrasonicated (15 min at the temperature of 0 °C) and centrifuged (5635× *g*, 10 min, 0 °C). Following this step, the supernatant was collected in dark vials. The Phenomenex Synergi Max-RP 80A column (250 × 4.6 mm) was used for compound separation. The injection volume was 100 µL and detection was performed under the wavelength of 445 and 450 nm for 18 min. The quantification of the compounds was based on external standards. Calibration curves were prepared individually for each of the compounds. For this purpose, pure standards were dissolved in acetone. Standard concentrations were as follows: *β*-carotene 20 µg/mL, α-carotene 5 µg/mL, and lutein 6 µg/mL. Each standard was injected five times—every time with doubled volume compared to the previous one. Standard curves were developed based on the outcomes of the described procedure.

### 3.3. Statistical Analyses

The main effects of the production system (organic vs. conventional) and the carrot cultivar (Flacoro vs. Nantejska) on the concentrations of the measured compounds, as well as the interactions between the two factors, were analyzed using 2-factor ANOVA, followed by the post hoc Tukey’s honestly significant difference (HSD) test (*α* = 0.05). The normality of the data was tested with a qqnorm function. No data transformations were necessary. The analyses were performed with the ‘nlme’ package in the R statistical program [48]. Means with standard deviations (SD) for the concentrations of the identified compounds in Flacoro and Nantejska carrot roots from the organic and conventional systems, together with the indication of statistical significance of the system-based and cultivar effects and their interactions (α = 0.05), are presented in Table 1. In addition, Pearson’s product-moment correlation analyses between concentrations of individual phenolic compounds and carotenoids were performed in R, and a correlation matrix figure (Figure 4) was produced using the ‘corrplot’ package.

## 4. Conclusions

This study has shown that the organic production system does not necessarily guarantee higher contents of such compounds as carotenoids and phenolics in carrots. Carotenoids concentrations were found to be higher in the conventional as compared to the organically grown carrot roots, while the measured phenolics concentrations have shown a large inter-sample variation. Additional research efforts could be encouraged to increase the likelihood that organic crops reaching the market would consistently contain the expected high levels of health-promoting bioactive compounds, which could be brought through their shelf-life and processing, in order to meet consumers’ expectations and provide the expected health benefits. Carrying out research that could potentially allow the identification and promotion of factors and/or strategies to enhance the nutritional and health-promoting qualities of vegetables cultivated in alternative, environmentally sustainable systems is highly relevant. As the carrot is a popular vegetable globally, and the consumption of carrots and carrot-based processed foods, including those organically produced, has been increasing steadily in recent years, this vegetable is a relevant target for such studies.

## Figures and Tables

**Table 1:** The effect of the agricultural production system (conventional and organic) and the cultivar (Flacoro and Nantejska) on the dry matter content (g/100 g fresh weight) and the concentrations of phenolics and carotenoids (mg/100 g) in the carrot roots studied (mean ± standard deviation).

	cv. Flacoro		cv. Nantejska		ANOVA *p*-Values		
	Conventional	Organic	Conventional	Organic	System (S)	Cultivar (C)	S × C
Dry matter	12.7 ± 2.2	13.5 ± 1.9	13.1 ± 0.8	13.9 ± 1.5	0.086	0.453	0.968
Polyphenols (sum)	7.69 ± 2.78	8.85 ± 3.13	9.35 ± 2.27	9.74 ± 3.21	0.392	0.272	0.638
Phenolic acids (sum)	3.91 ± 0.91	4.09 ± 1.27	4.60 ± 1.87	4.88 ± 1.38	0.580	0.175	0.904
Chlorogenic acid	1.49 ± 0.91	1.12 ± 0.67	1.17 ± 0.90	0.96 ± 0.74	0.193	0.402	0.705
Gallic acid	1.45 ± 0.24 ^bc^	1.79 ± 0.54 ^b^	1.14 ± 0.19 ^c^	2.38 ± 1.00 ^a^	**0.000**	0.250	**0.019**
Caffeic acid	0.26 ± 0.26	0.26 ± 0.23	0.32 ± 0.17	0.24 ± 0.21	0.427	0.873	0.518
*p*-Coumaric acid	0.71 ± 0.24 ^b^	0.93 ± 0.70 ^b^	1.97 ± 0.83 ^a^	1.30 ± 1.42 ^ab^	0.275	0.123	0.110
Flavonoids (sum)	3.78 ± 2.21	4.76 ± 2.13	4.75 ± 0.64	4.86 ± 2.23	0.374	0.516	0.431
Quercetin-3-*O*-rutinoside	0.56 ± 0.57	0.58 ± 0.43	0.58 ± 0.14	0.54 ± 0.30	0.943	0.854	0.790
Quercetin-3-*O*-glycoside	0.92 ± 0.52	0.88 ± 0.63	0.98 ± 0.39	0.72 ± 0.50	0.259	0.588	0.440
Kaempferol-3-*O*-glycoside	0.23 ± 0.19	0.52 ± 0.43	0.43 ± 0.62	0.44 ± 0.49	0.313	0.941	0.282
Luteolin	0.28 ± 0.18 ^b^	0.34 ± 0.20 ^b^	0.61 ± 0.4 ^a^	0.29 ± 0.22 ^b^	**0.029**	0.327	**0.010**
Apigenin	0.88 ± 0.41	1.39 ± 0.93	1.17 ± 0.68	1.66 ± 1.07	**0.045**	0.345	0.960
Keampferol	0.91 ± 0.57	1.05 ± 0.68	0.98 ± 0.56	1.21 ± 0.62	0.281	0.499	0.775
Carotenoids (sum)	4.22 ± 0.78 ^ab^	4.36 ± 0.70 ^ab^	5.00 ± 0.82 ^a^	3.84 ± 0.71 ^b^	**0.005**	0.751	**0.002**
Lutein	0.09 ± 0.01	0.09 ± 0.01	0.09 ± 0.01	0.09 ± 0.01	0.215	0.458	0.953
α-carotene	0.39 ± 0.14 ^ab^	0.37 ± 0.03 ^b^	0.41 ± 0.07 ^ab^	0.43 ± 0.08 ^a^	0.917	0.131	0.329
*β*-carotene	3.75 ± 0.63 ^bc^	3.90 ± 0.66 ^ab^	4.50 ± 0.76 ^a^	3.31 ± 0.63 ^c^	**0.002**	0.555	**0.001**

Values in the same row followed by different superscript letters (a–c) are significantly different at the 5% level of probability (Tukey’s HSD test). Significant ANOVA *p*-Values (*p* < 0.05) are marked in bold.

## Data Availability

Data will be made available upon a reasonable request to the corresponding author (Dominika Średnicka-Tober).

## Sample Availability

Samples of the compounds are not available from the authors.
